# Biochip for the Detection of *Bacillus anthracis* Lethal Factor and Therapeutic Agents against Anthrax Toxins

**DOI:** 10.3390/membranes6030036

**Published:** 2016-06-24

**Authors:** Vitalii Silin, John J. Kasianowicz, Ariel Michelman-Ribeiro, Rekha G. Panchal, Sina Bavari, Joseph W. F. Robertson

**Affiliations:** 1 Physical Measurement Laboratory, National Institute of Standards and Technology, Gaithersburg, MD 20899-8120, USA; vitalii.silin@nist.gov (V.S.); john.kasianowicz@nist.gov (J.J.K.); lasirenita54@yahoo.com (A.M.-R.); 2 NIST Center for Neutron Research, National Institute of Standards and Technology, Gaithersburg, MD 20899-8120, USA; 3 Institute for Bioscience and Biotechnology Research, University of Maryland, Rockville, MD 20899, USA; 4 US Army Medical Research Institute of Infectious Diseases, Fort Detrick, Frederick, MD 21702-5011, USA; rekha.g.panchal.civ@mail.mil (R.G.P.); sina.bavari.civ@mail.mil (S.B.)

**Keywords:** anthrax, protective antigen, lethal factor, edema factor, therapeutic agents, screening, tethered bilayer membrane, biochip

## Abstract

Tethered lipid bilayer membranes (tBLMs) have been used in many applications, including biosensing and membrane protein structure studies. This report describes a biosensor for anthrax toxins that was fabricated through the self-assembly of a tBLM with *B. anthracis* protective antigen ion channels that are both the recognition element and electrochemical transducer. We characterize the sensor and its properties with electrochemical impedance spectroscopy and surface plasmon resonance. The sensor shows a sensitivity similar to ELISA and can also be used to rapidly screen for molecules that bind to the toxins and potentially inhibit their lethal effects.

## 1. Introduction

The development of biosensors often requires the attachment of fully functional proteins to solid surfaces [1,2,3,4]. The use of integral membrane proteins for this application is particularly challenging, because they require a nanometer-scale hydrophobic environment opposed by two aqueous phases. To address this issue, several types of supported bilayer lipid membranes (SBMs) were developed [4].

The first generation of SBMs relied on the direct adsorption of lipid, through vesicle rupture, to a hydrophilic surface (e.g., silica). SBMs are an important tool for both the study of membrane proteins and sensors that rely on membrane proteins [1,5]. They have also been used extensively to study lipid membrane properties and molecular interactions between bilayers [6]. Because the membrane is adsorbed directly onto the substrate, this technique generally only accommodates proteins that do not fully span the bilayer. The use of cytoskeletal s-layer proteins, in place of silica as the support, increases the SBM stability [7,8,9,10]. However, to date, SBMs with protein supports have only been able to accommodate small membrane proteins (i.e., gramicidin has been shown to insert, but alpha-hemolysin does not) for biosensing applications [10].

In an attempt to make supported membranes with improved mechanical stability and low ionic conductance, hybrid bilayer constructs were developed [11]. Hybrid membranes were initially made by adding a lipid monolayer atop a self-assembled monolayer (SAM) comprised of alkanethiols covalently linked to the support [11]. However, the proximity of an alkanethiol to the solid surface and the membrane’s lack of fluidity can significantly compromise the reconstitution of membrane-spanning proteins [12].

Cornell and colleagues demonstrated that replacing single chain fatty acids in the SAM with lipid mimics enabled the development of a biochip that can make use of a fully functional membrane pore-forming protein [13]. This class of tethered bilayer lipid membranes (tBLMs) is characterized by an inner monolayer comprised of a membrane anchor molecule with three components: a lipophilic moiety that forms half the bilayer, a hydrophilic polymer spacer (e.g., poly(ethylene glycol), DNA, peptide), and a surface binding component (e.g., thiol, chlorosilane) [14,15]. Early versions of tBLMs used a self-assembly cocktail that both anchored the membrane and channel forming units (i.e., proteins or peptides) [13,16]. Later developments introduced pure compounds that more closely resemble natural lipids, albeit with unnatural head groups [17,18], and two component mixtures of thio-lipids with small molecules [19,20,21], thiol-linked cholesterol [22] or even proteins themselves [23,24]. In practice, the nature of the anchoring molecule, lateral spacing of the anchoring molecule on the surface, and membrane composition depends on the application [25].

tBLMs were originally developed as an electrochemical biosensor with a transmembrane protein as both the signal transducer and part of the recognition element [13]. Many tBLM-based sensors have been developed based on membrane proteins, including small pore-forming peptides such as gramicidin [13,19,26] and mellitin [19], large oligomeric bacterial toxins such as alpha-hemolysin [27,28], membrane protein fragments such as influenza virus matrix protein 2 (M2) [29], and redox proteins (e.g., cytochrome C oxidase [23,24], cytochrome *bo*_3_ [30] or CymA [31] and many others) [32].

The amphipathic nature of membrane proteins make them challenging to assemble into tBLMs [33]. In the simplest case, water-soluble or detergent-solubilized proteins, injected into the solution bathing the tBLM, spontaneously partition into the membrane. Solubility is, however, not a requirement. Recent work suggests that in vitro transcription and translation can also be used to synthesize, fold and insert some proteins directly into a tBLM [34,35].

The detection of biological toxins and screening for potential therapeutic agents against them are critical public health issues [36]. Many sensing schemes have been developed to detect and characterize biological toxins [37]. Usually, the biological recognition element (e.g., an enzyme, receptor, antibody) is immobilized onto an interface (e.g., a sensor surface) and is interrogated either an electrochemically or optically [37]. In this paper, we describe a biosensor for the direct detection of *B. anthracis* toxins that are competitive with ELISA-based assays [38].

*B. anthracis* secretes three separate proteins, which constitute an AB toxin. The AB toxin motif is common among pathogenic bacteria from *Bacillus* and *Clostridium* species [39,40]. The “A” is an activity component, which is often an enzyme that catalyzes cell death, while “B” is a binding component with interacts with receptors on a cell membrane and often chaperones the “A” component into the cell. In *B. anthracis*, the “B” component (protective antigen) is secreted as an 83 kDa protein which is cleaved into a 63 kDa activated protein (PA63). PA63 forms heptameric [41] and octameric [42] pores which bind the “A” component (i.e., Lethal Factor, LF, and Edema Factor, EF) and chaperone them into cells [40,43]. We previously demonstrated that a simple instantaneous current-voltage measurement of *B. anthracis* PA63 ion channels in unsupported black lipid membranes (BLMs) provided a sensitive and rapid assay for the detection of *B. anthracis* toxins (i.e., Lethal Factor, LF, and Edema Factor, EF) and potential therapeutic agents [44]. Similar studies showed that small molecules can prevent LF or EF binding and inhibit anthrax toxin efficacy [45,46,47]. Because classical BLMs are not sufficiently robust for use in a clinical setting, we wanted to determine whether a tBLM doped with PA63 channels could serve as a biochip to detect anthrax toxins. The tBLM offers superior structural stability and can be maintained in solution for months without showing signs of degradation [48]. That stability makes biochips with this architecture a promising support for a clinical biosensor. This report summarizes the fabrication and characterization of a PA63-based tBLM biosensor and demonstrates the potential use of the electrical signal from electrochemical impedance spectroscopy (EIS) and the optical signal from surface plasmon resonance (SPR) as complementary detection modalities.

## 2. Results

### 2.1. Biosensor Fabrication

**Figure 1:**
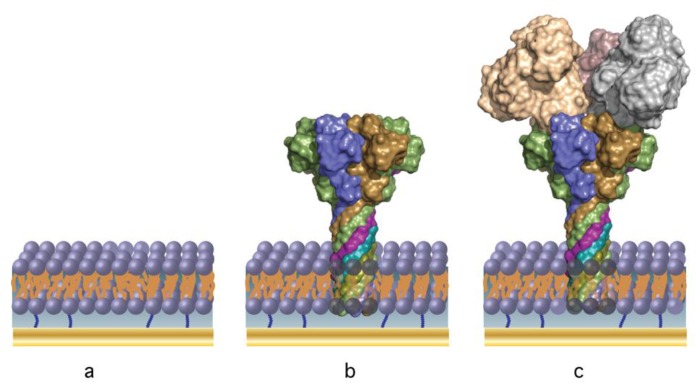
The anthrax biochip built from the sequential deposition of (**a**) a tethered bilayer lipid membrane, and (**b**) self-assembling *B. anthracis* PA63 ion channels (nanopores) [41]—the selective sensing element; (**c**) *B. anthracis* Lethal Factor [53] is detected by binding to the PA63 channel cap domain [43] via electrochemical impedance spectroscopy and surface plasmon resonance.

Figure 1 shows an idealized structure of the tBLM-supported PA63 biochip. Initially a self-assembled monolayer (SAM) is created ex situ by incubating a freshly prepared Au surface (see Section Materials and Methods) in an ethanolic solution of thio-lipid (the anchor) and a diluent (β-mercaptoethanol). The ratio of thio-lipid to diluent controls many of the membrane properties (e.g., fluidity, stability) and the ability to reconstitute proteins [48,49]. As a compromise between protein coverage and membrane stability, we work exclusively with a surface assembled from a solution mixture of 30% WC14 (see materials and methods for details) and 70% βME, [20,21,50]. The membrane is formed via rapid solvent exchange [20], resulting in a conformal, defect-free bilayer as shown in Figure 1a.

Once the membrane is formed, PA63 is injected into solution, which spontaneously adsorbs to the membrane surface and forms ion channels (Figure 1b). The pore provides the means to detect the enzymatic components of anthrax toxins (such as Lethal Factor, LF and Edema Factor, EF) [44], as they bind at high affinity to the channel cap domain [40,44,51,52] (Figure 1c). EIS and SPR are used to monitor the assembly of the biochip and as the sensing modalities.

### 2.2. Electrochemical Impedance Spectroscopy

**Figure 2:**
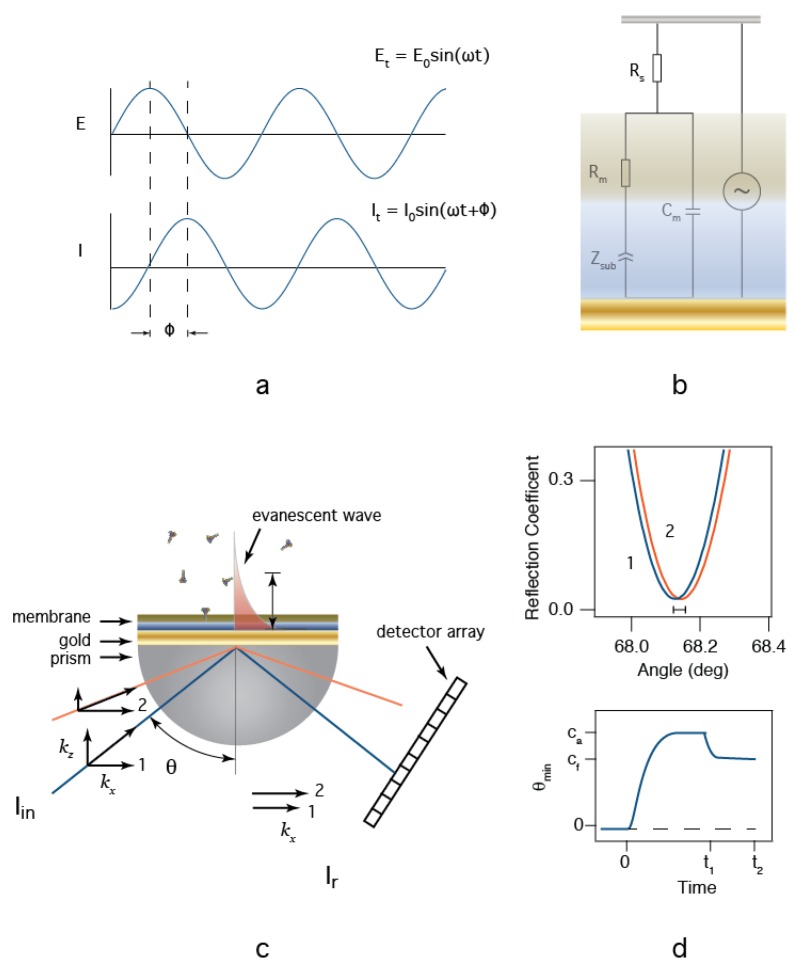
Electrochemical impedance spectroscopy (EIS) and surface plasmon resonance (SPR) provide orthogonal measurements for the study of membrane protein-based biochips. (**a**) In EIS, a sinusoidal potential difference is applied between the reference and working electrodes. The current is monitored and impedance modulus (*E*/*I*) and phase shift (ϕ) are recorded as a function of frequency. (**b**) EIS data is analyzed by fitting the data to an equivalent circuit model (*see text for details*); (**c**) SPR measurement schematic. Light is coupled into the plasmon modes of an ultra-thin Au film through a high index of refraction prism, and (**d**) the reflected light intensity is recorded (*top*). The angle at which the reflected light intensity reaches a minimum is observed as a function of time as analyte is added at *t* = 0, and washed from the cell at *t* = *t*_1_ (*see text for details*).

EIS is an electrical method used in the study of complex interfaces [54]. Figure 2a illustrates an AC electric potential (*E*) applied between the reference electrode (in the aqueous solution) and the working electrode (the tBLM-modified Au surface) and the resultant ionic current (*I*). The amplitude of the current, and the phase difference between the applied potential and current are measured. In a typical EIS experiment, the frequency is varied over orders of magnitude, which allows for the separation of the characteristic time constants associated with mobile ions moving in response to the oscillating potential. The data is then either analyzed directly or fit with a model equivalent circuit [55,56]. We apply the circuit model developed by Valincius (Figure 2b) [50] to approximate the physical parameters of our sensor. In this model, *R_s_* is the solution resistance in series with the membrane:nanopore interface. This interface is described by an interfacial capacitance (*C_m_*) in parallel with the membrane resistance (*R_m_*) and the complex impedance of the tether:aqueous region between the membrane and the Au surface (*Z_sub_*). The interpretation of *C_m_* is the series combination of the membrane capacitance and the electrochemical double layer. The resistive pathway is more complicated. *R_m_* is the net resistance of the membrane, water-filled defects in the membrane, and protein ion channels. Each of these components will be in parallel with each other and add reciprocally. The remaining element (*Z_sub_*) is less intuitive. We follow the convention outlined earlier [50,56], where the complex impedance in the tether is modeled as a constant phase element (CPE) with the form $Z_{sub}=1/\left[T{\left(i\omega \right)}^{P}\right]$ [55], where, ω is the applied frequency in rad/s, *T* is a scaling parameter with units of S·s^P^, and *P* is an unitless exponent that scales between 0 and 1. At the extremes, *Z_sub_* reduces to a resistor when *P* = 0 and a capacitor when *P* = 1. Utilizing the CPE in such a manner allows the pores to be treated as discrete resistors by providing a conductive pathway through the hydrated tether. Curve fits of equations to the impedance modulus (|*Z*| = *V*/*I*) and phase angle data provide measurements for the circuit element values. In the application described herein, this model is used to determine the values for *R_m_*, which is a direct measurement for the presence of other anthrax toxins and the effectiveness of therapeutic agents against them. To estimate the number of nanopores inserted into the membrane, we assume that *R_m_* is shorted by discreet non interacting pores with a resistance *R_p_*. Thus, $R_{m}^{-1}=R_{m,\;t=0}^{-1}+nR_{p}^{-1}$, where *R_m_* is the measured membrane resistance after protein is added, *R_m,t =_*_0_ is the measured membrane resistance before protein addition, *R_p_* is the resistance of a single pore and *n* is the total number of pores.

### 2.3. Surface Plasmon Resonance

Surface plasmon resonance (SPR) is an optical technique extensively used to detect the adsorption of molecules to surfaces in real-time [57]. In a biosensor, the surface is typically modified with an adhesion element (e.g., a protein) that is selective for the analyte of interest [58]. Surface plasmons (SP, i.e., surface electromagnetic waves) are a combined state of electromagnetic wave and charge density of surface electrons. SP are excited by incident photons at interfaces where the dielectric constants of the media at the interface have opposite signs, which occurs at many metal-solution interfaces. While Au and Ag are the two most common metal films used in SPR [57,58], Au is often the metal of choice, because it is chemically inert and amenable to surface modification with thiol chemistry. In this work, we use the Kretchmann configuration [57,58] in which light is coupled into the device, (e.g., via high index of refraction prism), made incident on a thin semitransparent Au film deposited on the prism’s bottom (or optically coupled to an index of refraction matched glass slide coupled with an index matching oil), and reflected onto a photodetector array. The instrument used in this work used a diode light source that is focused at a reflection point on the surface. Reflected light is detected on an array (i.e., camera), where the angle of reflection is estimated by monitoring the light intensity as a function of pixel position. To create the resonance condition necessary to excite an SP, the wave vector of the incident photons must match the SP’s wave vector. Because the wave vector, *k_sp_*, propagates in the *x*-direction from the point of incidence, resonance is achieved when the *x*-component of the incident light wave vector, *k_x_* = *k_sp_* (Figure 2c). Altering the angle of incidence changes *k_x_*. When the resonance condition is met, light is absorbed into the SP, creating a minimum in reflectivity (Figure 2d *top*). The resonance conditions that produce a minimum reflectivity at a particular angle are extremely sensitive to the optical properties of interfacial media at and above the Au surface. Thus, by measuring changes in the resonance angle during biomolecule adsorption within the evanescent wave adjacent to the Au film surface, subtle changes to the nanometer-scale layers of biomolecules at the biosensor surfaces can be monitored.

For the biosensors prepared here, we estimate the expected SPR response using [59,60], which solves the Fresnel equations for a four layers interface. The resonance curves in Figure 2d were calculated for with a high index of refraction prism, a 50 nm thick Au layer as the base with a protein-free tBLM in contact with electrolyte buffer (*blue*) when ~1% of the surface is covered with a ~10 nm thick protein film (*orange*) the resonance minimum shifts according to the increased optical thickness. This calculation suggests that we should observe a shift in the minimum angle ~0.02 degrees for every 1% surface coverage of protein (Figure 2d *top*). This shift can be expressed as an optical thickness, which is the thickness of the layer times the difference in refractive indices of the film and bulk media. Thus, with our instrument calibrated to 1 pixel on the camera equal to 0.647 Å, we can directly estimate the amount of material adsorbed during an experiment. These changes are followed over time in a manner depicted in Figure 2d *bottom*. In a typical experiment, protein is injected into the cell at *t* = 0 and an adsorption phase is observed as a shift to larger angles. For detailed binding constants to be measured, the experiment is followed until the signal reaches a steady state (c_a_) suggesting that an equilibrium is achieved. At *t*_1_, the analyte (protein) is washed out of the cell and a desorption phase is observed where excess weakly bound material is removed from the surface. Because the LF:PA63 interactions used for the sensor in this paper are essentially irreversible, no attempt is made to determine binding constants from the SPR data. The sensitivity of the measurement routinely allows for the detection of sub-monolayer amounts of material to be detected [57,58]. Because the SP is typically excited from the backside of the surface of interest, SPR can be combined with other techniques, such as EIS.

### 2.4. *B. anthracis* Toxin Biochip Formation

**Figure 3:**
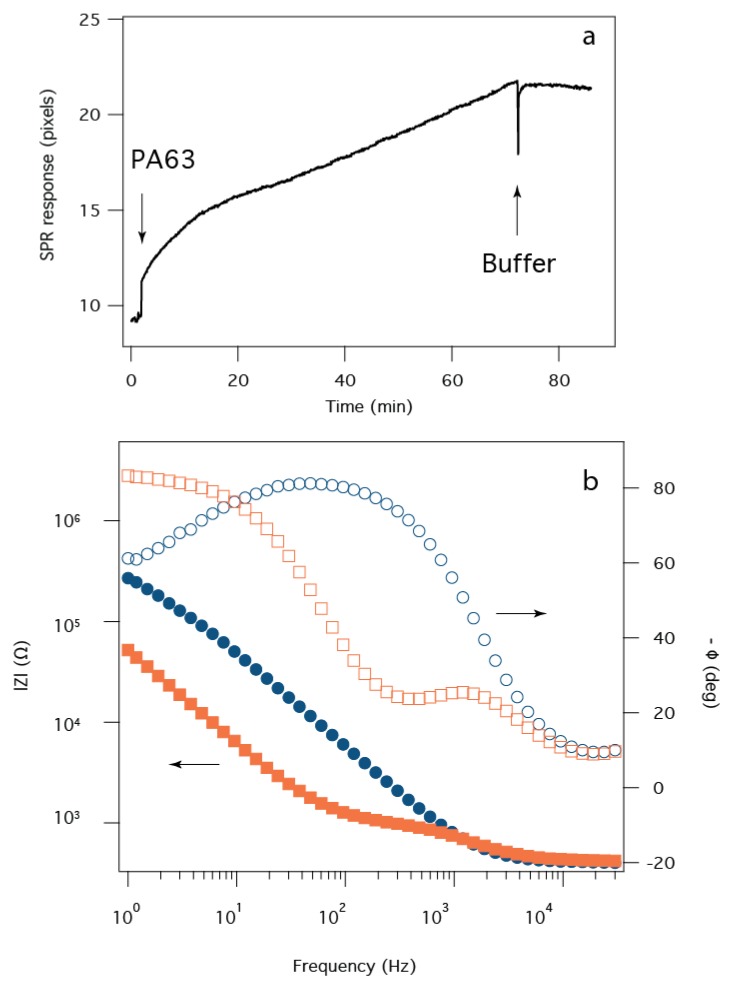
Assembly of the sensor shown monitored with SPR and EIS. (**a**) SPR time series for the adsorption of PA63 onto the tBLM after injection to 320 nM into the subphase at *t* ~ 0. The response corresponds to PA63 adsorbing to the tBLM; (**b**) The EIS data for the tBLM only (*blue circles*) and the tBLM after PA63 incorporation into the membrane (i.e., >80 min in (**a**) (*orange squares*). The pH of the solution is pH 6.6.

Development of anthrax toxin biosensor chips requires precise control over PA63 channel formation in tBLMs. An SPR sensorgram time series (Figure 3a) shows that the addition of PA63 to the aqueous solution causes a two-phase increase in the SPR minimum angle. At the first arrow, PA63 is added to a bulk concentration of 320 nM. Initially (*t* < 15 min), PA63 adsorbs to the surface relatively rapidly, but the rate decreases thereafter. The initial rate corresponds to the adsorption of fully-functional PA63, while the lower rate most likely is due to a decrease in active (i.e., pore forming) PA63 in bulk solution (*data not shown*). Subsequent removal of excess PA63 with fresh electrolyte buffer solution causes the SPR signal to stabilize, as expected.

The corresponding EIS data shows a highly resistive bilayer membrane prior to protein injection (Figure 3b, *blue circles*), with *R_m_* > 200 kΩ. After the protein is added to the solution and subsequently flushed from the cell, the decrease in |*Z*| and the relatively narrow phase angle minimum at a frequency of *f* ~ 300 Hz corresponds to a membrane resistance *R_m_* ~ 10^3^ Ω. This marked decrease of >10^5^ Ω can be attributed to ~10^5^ PA63 nanopores (assuming the conductance is ~80 pS/channel [44]) in the tBLM that has a surface area ~0.3 cm^2^, which suggests the pores occupy only ~10^−6^% of the available surface area.

### 2.5. Detection of *B. anthracis* Toxin LF

**Figure 4:**
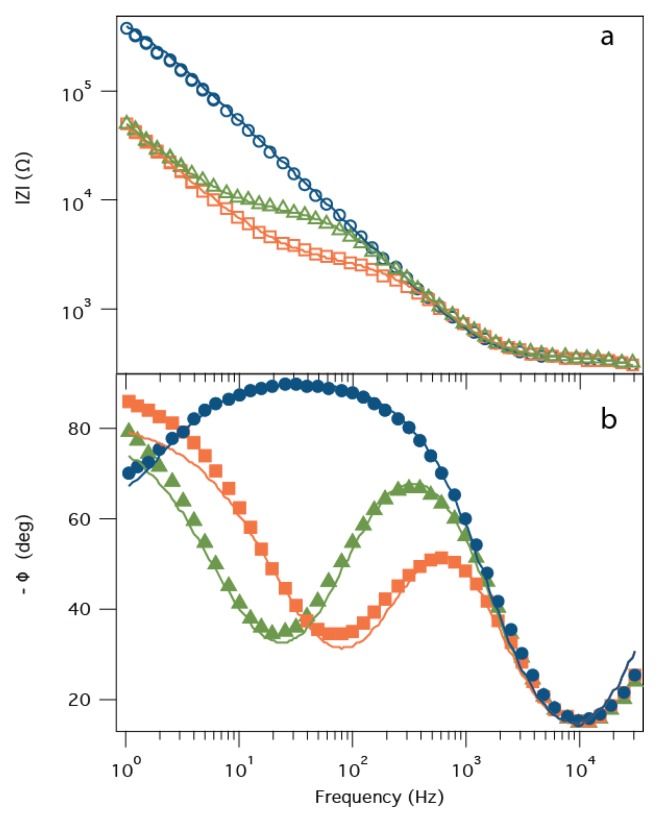
Detection of LF with the PA63:tBLM biochip via EIS. The impedance (|*Z*|) (**a**) and phase angle (ϕ); (**b**) as a function of frequency for the tBLM only (*blue circles*), PA63:tBLM (*orange squares*), and the tBLM:PA63 biochip + 1 nM *Bacillus anthracis* LF (*green triangles*). Solid lines are non-linear least squares fits of the model presented in Figure 2b to the data.

With its large cap domain, the PA63 channel should reconstitute in the tBLM with the same orientation as other pore forming toxins such as α-hemolysin [28], i.e., with the LF binding site (in the channel’s cap domain) on the bulk solution side of the membrane. Previous work demonstrated that when LF binds to the PA63 channel, the instantaneous *I*-*V* relationship measured with a DC voltage clamp setup changes from nearly linear to extremely rectified [43,44]. Specifically, the current obtained with negative applied voltages was virtually unchanged, but markedly decreased for positive potentials. In the PA63:tBLM biochip, LF binding to the PA63 channels should increase *R_m_*, leading to a decrease in the frequency of the phase angle minima and an increase in *Z* in the frequency range at which the phase minimum occurs [56]. Figure 4 shows precisely this behavior. Initially, a high impedance membrane (*R_m_* > 800 kΩ) is incubated with PA63 in the bulk aqueous solution and *R_m_* decreases to ~3 kΩ. The addition of 55 pM LF to the measurement cell increases *R_m_* to ~10 kΩ, consistent with LF blocking some of the PA63 channels in the tBLM [44]. Under the conditions where this sensor operates (i.e., low protein coverage) and pH between ~5.5 and ~7.2 [61], the values for *C_m_* and *Z_sub_* are essentially constant, at *C_m_* ≈ 0.3 μF and *Z_sub_* (T) ≈ 3.5 μS·s^P^ and *Z_sub_* (P) ≈ 0.9. In the absence of pores, *Z_sub_* is not a significant contributor to impedance of the system and is poorly fit. While the model fits the data satisfactorily, the deviations seen, particularly in the low frequency phase shift, suggest that the equivalent circuit does not fully describe the ion movement in the tether-region of the tBLM.

**Figure 5:**
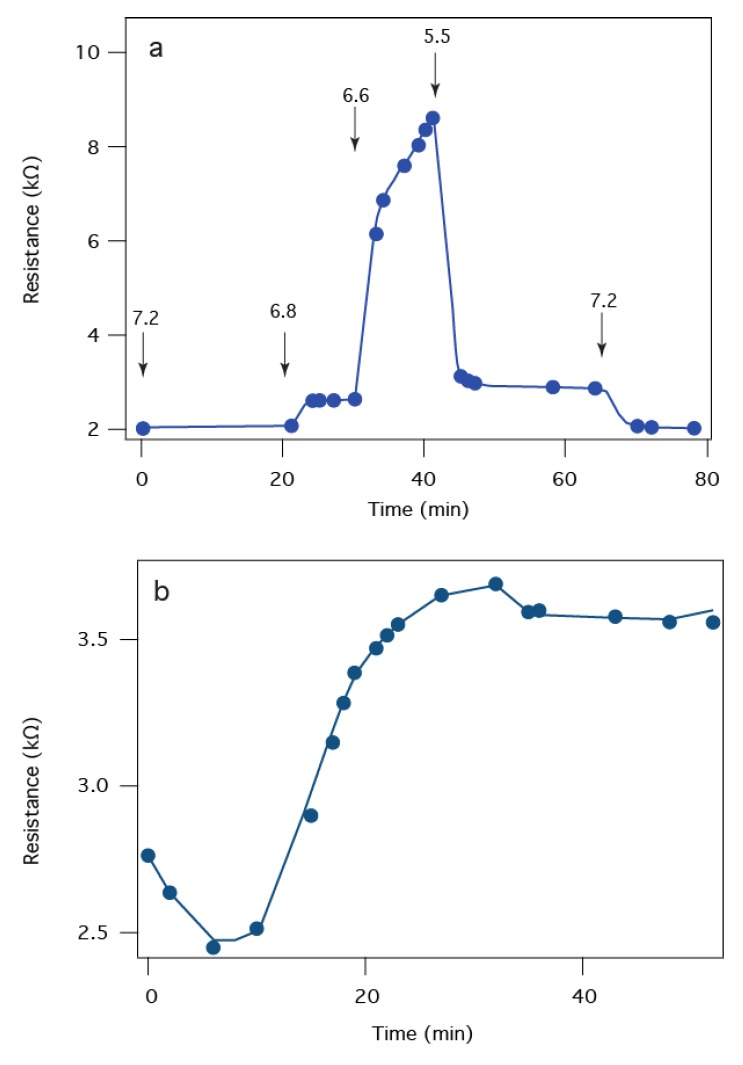
LF binds to a PA63:tBLM biochip. (**a**) The magnitude of the LF-induced resistance change is pH dependent. The arrows indicate the time at which the pH was adjusted to the indicated values with 10 nM LF present in each case; (**b**) The biochip EIS response to 50 pM LF added at *t* ~ 5 min.

Figure 5 illustrates more details on the electrochemical sensor response of the PA63:tBLM biochip to LF. Previous studies indicates that the interactions between LF and PA63 is pH dependent [43,62]. Figure 5a shows the sensor response to an injection of 10 nM LF as the pH is changed. Initially, at pH 7.2, a resistance of ~2 kΩ is observed (from a 1 kΩ baseline), which suggests the LF present in that solution did not block the PA63 channels. As the solution is stepped to pH 6.8, the resistance increases slightly. At pH 6.6, a much larger resistance increase occurs, which suggests LF is more effective at blocking the PA63 channel conductance, as expected [44]. At pH 5.5, the resistance abruptly drops. At the end of the measurement, the pH is returned to pH 7.2 and the initial 2 kΩ signal is recovered indicating no changes in the PA63 surface during the titration. The physical basis of this pH dependence of LFs effect on the PA63 channel conductance is unclear. It has been suggested that acidification can drive LF to cross the channel through the PA63 pore [62] although other evidence suggests that if the solution is acidified in the presence of LF in solution the binding is irreversible [43]. In the tBLM chip, LF cannot cross the membrane due to insufficient space in the tether region, so the resistance changes are likely due to changes in the LF:PA63 interaction. Regardless of the mechanism, the biochip’s peak sensitivity is achieved at pH 6.6. Figure 5b shows that the PA63:tBLM biochip has similar sensitivity as PA63 channels in BLMs with a clear irreversible binding at all pH values tested here (i.e., <pH 6.6) interaction at 50 pM LF. Negative control experiments (SPR and EIS) show no specific interaction of LF with a PA63-free membrane up to concentrations in excess of 500 nM, well beyond the ~15 pM to 600 pM concentrations found in patients diagnosed with inhalation anthrax [63].

Our previous work shows that the binding constant of LF to PA63 is between 20 pM at pH 7.2 and 40 pM at pH 6.6—assuming 1:1 stoichiometry [43,44]. The binding data is complicated due to the partial denaturation of LF under acidic conditions [64], which rebinds to the PA63 pore irreversibly [43]. The toxin complex can also be observed intact in blood from infected animals [65] suggesting that the dissociation constant is immeasurable. The structure of these complexes have been observed with electron microscopy [66], and studied with computer simulations [42,67] to confirm the strong and specific interaction of PA63 pores with LF. Electron spin resonance experiments suggest that acidification of the solution causes the N-terminal segment of LF to interact strongly with a region inside the pore [51], which is consistent with the increase in resistance down to pH 6.6. The mechanism for the sharp decrease in resistance at pH 5.5 is unclear. To fully understand the pH dependent sensitivity of the presented in Figure 5, additional high-resolution structural data will be necessary.

**Figure 6:**
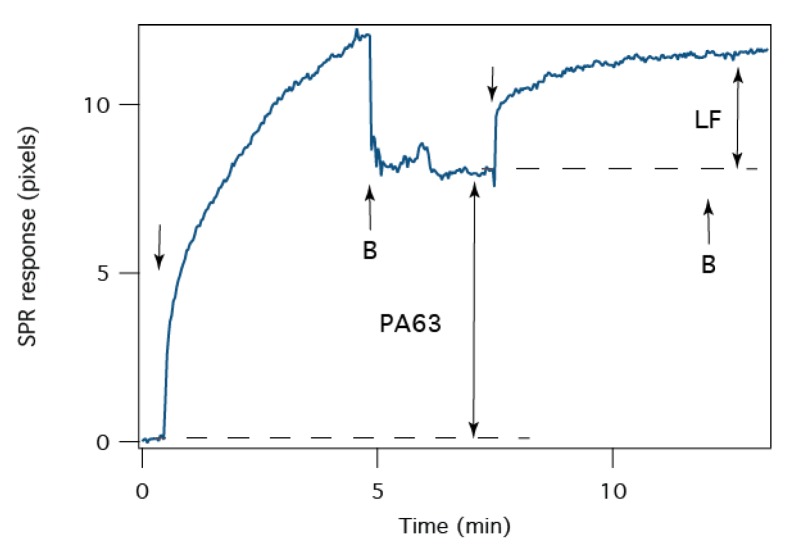
The average stoichiometry of the PA63 channels and LF determined by SPR at pH 6.6. The arrows indicate where PA63- or LF-containing electrolyte solution is added to the cell. The arrows marked “B” indicate when PA63- or LF-free electrolyte solution is added. The double-headed arrows indicate where the SPR signal was used to determine the relative coverage of PA63 and LF. The buffer exchange was removed from the time series for clarity.

The stoichiometry of the PA63:LF interaction is monitored with SPR. The sensorogram time series in Figure 6 shows the adsorption of PA63 to the tBLM. After ~5 min, adding fresh buffer removes PA63 from the cell, and the net SPR response is ~7.8 pixels at *t* ~ 7 min, LF is injected into the cell. The binding of LF to PA63 in the tBLM, which further shifts the SPR minimum by 3.6 pixels. In the simplest application of SPR analysis, the resonance shift is directly proportional to the adsorbed mass of analyte [58]. Assuming that the PA63 in the tBLM is in the heptameric form (i.e., 441 kDa/channel), LF is monomeric (90 kDa), and the proteins have the same density, the SPR data suggests that, on average, there are 2 LF molecules bound to each channel.

The stoichiometric data presented here is offered as a control. The ionic current pathway was shown to be blocked with 1:1 LF:(PA63)_7_ binding [44], but the SPR data can sense additional binding events. The heptameric PA63 channel cap domain can accommodate up to three LF molecules [52,68]. The number quoted in the text is the mean value over all possibilities and should follow a Poisson distribution.

### 2.6. Screening of Therapeutic Agents against *B anthracis* Toxins

**Figure 7:**
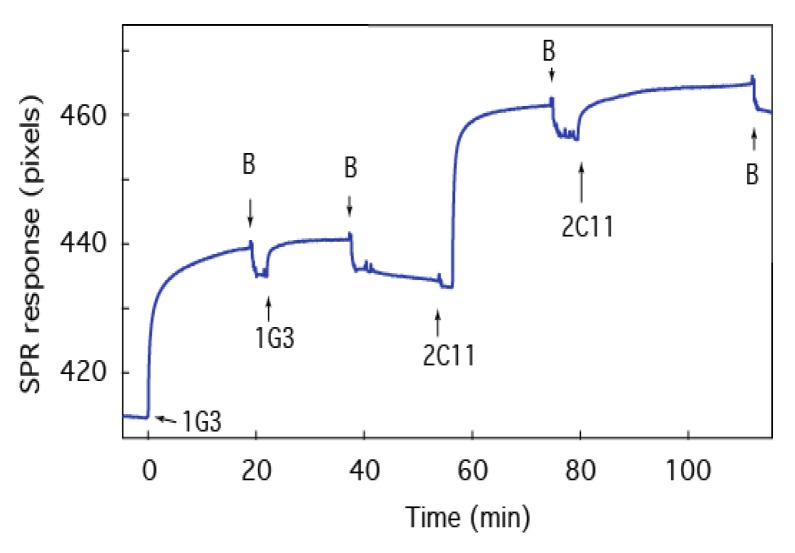
Anthrax therapeutic agents bind to the biochip. SPR time series of the tBLM:PA63 biochip after adding antibodies 1G3 and 2C11 to the cell. The arrows labeled B indicate the removal of excess antibodies with fresh electrolyte solution. The electrolyte is 0.1 M KCl buffered with 10 mM MES at pH 6.6.

One particular advantage of the PA63:tBLM biochip platform is the ability to use several detection methods for biomolecules. For example, monoclonal anti-PA63 antibodies 1G3 did not change the instantaneous *I*-*V* response of PA63 channels in unsupported BLMs taken shortly after the antibody was added [44], which could limit the effectiveness of an electrochemical sensor. SPR provides an orthogonal means of detecting the antibody-PA63 interaction (Figure 7). Many anti-PA63 monoclonal antibodies have been developed [69,70,71,72,73], so having a method that could rapidly screen for those that bind strongly to the PA63 in a membrane and possibly interfere with anthrax toxin deleterious effects would be imminently useful.

To test the antibody response, a PA63:tBLM biochip is incubated with anti-PA63 1G3 [72,74] in two successive steps and then anti-PA63 2C11 [75]. Upon injection of 1G3, the SPR signal shift increases, which suggests the antibody adsorbs to PA63 in the biochip (Figure 7). The addition of antibody-free solution (at the arrows labeled “B”), which washes out excess antibodies, has only a minor effect on the SPR signal, which suggests the antibody binds strongly to PA63. A second injection of antibody shifts the signal to the same maximum and subsequent buffer exchange returns the signal to the baseline of the bound complex, which suggests the interactions between the antibody and the PA63 are essentially irreversible and the binding sites for 1G3 are saturated. Interestingly, the same results were obtained with the subsequent addition of antibody 2C11. These experiments suggest that the two antibodies interact with different domains of the PA63 channel’s cap domain [76] and can be characterized sequentially on a single sensor surface.

**Figure 8:**
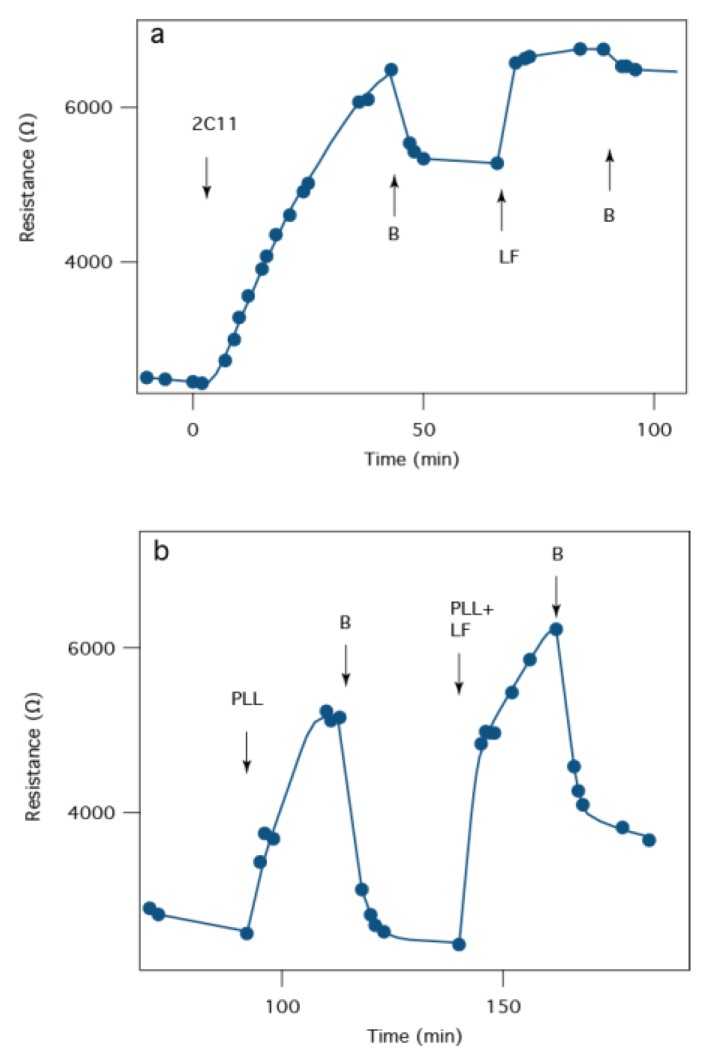
EIS response to the anthrax 2C11 antibody or a “confederate” molecule (poly-l-lysine, PLL) that might confound interpretation of the biochip results. (**a**) tBLM:PA63 biochip resistance time series in response to the addition of the 2C11 antibody (removal of excess 2C11 indicated at the arrows labeled B. After antibody incubation, LF was added at LF and washed from solution at B; (**b**) 10 nM PLL is injected into the cell and washed from the cell at the first “B” arrow. PLL and LF are subsequently co-injected at a concentration of 10 nm each at the third arrow and washed from the cell at following “B” arrow.

Although instantaneous *I*-*V* curves for PA63 channels do not show an ionic conductance change for 1G3:PA63 complex [44], EIS can be used to observe the reaction of antibodies on a PA63:tBLM biochip. For example, Figure 8a shows that 2C11 slowly increases *R_m_* over the course of >40 min, with no sign of reaching an asymptote. While the mechanism for the antibody-induced change in *R_m_* is not known, it is notable that the SPR signal reaches 90% of its maximum values after ~5 min (Figure 7), but *R_m_* is still changing after 30 min (Figure 8a). This discrepancy suggests that the antibody first binds to the pore and then the pore-antibody pair undergoes a conformation change (or relaxation), which either partially blocks the PA63 pore, alters the β-barrel dimensions or pulls the β-barrel out of the membrane.

Non-specific interactions must not confound the signal in a robust clinical sensor. As a control the sensor was challenged with positively charged poly-l-lysine (PLL), which is known to electrostatically interact with negatively charged side chains in the PA63 channel pore [43]. When PLL is added to the measurement cell, *R_m_* increases, as expected because it blocks the pore’s ionic conducting pathway (Figure 8b). However, unlike the specific and essentially irreversible binding of the two antibodies or LF to the PA63:tBLM biochip, PLL is completely removed from the surface when the excess is flushed from the bulk solution. When PLL is introduced with LF, *R_m_* increases to a greater extent, which suggests either that individual pores partially blocked by PLL are further blocked by LF, or some pores are not blocked by PLL but are blocked by LF.

## 3. Discussion

The results described here demonstrate the assembly and use of an anthrax toxin biochip that could serve as both a clinical sensor (i.e., with further development for the detection of *B. anthracis* toxins from blood or serum) and for screening potential therapeutic agents for anthrax treatment. The sensing scheme offers two orthogonal methods for detection: SPR and EIS. Because these methods are sensitive to different phenomena, additional controls for the sensor can be devised. In particular, the detection of pore forming channels, and molecules that bind to the pore can be separated from non-specifically bound species.

We also used EIS to estimate the biochip *receiver operating characteristic* for the detection of *B. anthracis* LF. For example, we determined the sensitivity of the PA63 chip to LF (<50 pM), and the response time of the system (minutes). Furthermore, we tested for one potential failure mechanism by detecting LF in the presence of a cationic polymer confederate (*i.e.,* PLL). The PLL does not confound the sensor for several reasons. First the PLL likely interacts electrostatically with the highly negatively charged pore interior [77,78], whereas LF binds (initially) to a different site that is on the channel’s cap domain [51]. Second, the PLL:PA63 interaction is reversible, with complete desorption observed in minutes, while LF apparently binds essentially irreversibly under the conditions used here. We also determined that two monoclonal antibodies (1G3 and 2C11), research tools that could potentially act as therapeutic agents against anthrax infection, bind strongly to PA63 pores on the tethered membranes. Antibody 2C11 does not appear to block LF binding to the membrane, which is consistent within vivo measurements of LF activity [76].

In addition to a functional anthrax biosensor, this study and others [27,35,79,80,81] demonstrate capabilities of a tBLM-supported biosensor using large membrane spanning proteins as the sensing element. In particular, we demonstrate that AB toxins such as the LF:PA63 from *B. anthracis* [40] provide a highly specific, built-in sensing scheme. This method should be easily extendible to other AB toxins found in such species as *Clostridium botulinum* [82], *Clostridium difficile* [39], *Clostridium perfringens* [83], and *Clostridium spiroforme* [39] and others.

## 4. Materials and Methods

### 4.1. Electrolyte Solutions and Analytes

Different electrolyte solutions were used to determine the effect of pH and buffer type on PA63 channel activity in the PA63:tBLM. The aqueous solution contained 0.1 M KCl, and 5 mM of either 2-(N-morpholino) ethanesulfonic acid (MES, pK_a_ = 6.15) 3-(N-morpholino) propanesulfonic acid (MOPS, pK_a_ = 7.20), citric acid (pK_a_ = 3.15, 4.77, 6.40), Bis-Tris propane (CABTP, pK_a_ = 6.8) obtained from Sigma-Aldrich (St. Louis, MO, USA). For all electrolyte solutions, deionized 18 MΩ water from a Millipore (Billerica, MA, USA) UHQ reagent-grade water purification system was used. Anthrax toxins PA63 and LF were obtained from List Biological Labs, Inc. (Campbell, CA, USA). Poly-l-Lysine (PLL) 28 kg/mol was obtained from Sigma-Aldrich. Activated PA63 and LF were obtained from List Biological Labs, Inc. 

### 4.2. SPR-EIS Measurements

For simultaneous SPR-EIS measurements, a custom-built SPR device was used. Measurements were carried out in a non-flow regime, using a 1 mL cell with a diameter of 6.5 mm. The SPR sampling area was 6 mm long and 40 μm wide. An LED light source (wavelength 650 nm) was used. The sensitivity of the device was ~10^−7^ units of refractive index. The dynamic range was between 1.32 to 1.41 units of refractive index.

For EIS measurements, we used either a Modulab electrochemical system or a model 1287 potentiostat and model 1252A frequency response analyzer and software (Solartron Analytical, Farnborough, Hampshire, UK). The spectra were obtained with a 10 mV AC potential for frequencies between 1 to 6.5 × 10^4^ Hz with 10 logarithmically distributed measurements per decade. The maximum rate of impedance sampling was up to one spectrum per 15 s. The reference electrode was a saturated silver-silver chloride (Ag/AgCl/NaCl (aq,sat)) microelectrode (M-401F, Microelectrodes, Inc., Bedford, NH, USA), and the counter electrode was a 0.5 mm diameter platinum wire (Aldrich, St. Louis, MO, USA, 99.99% purity) coiled around the barrel of the reference electrode. All measurements were carried out at 0 V direct current bias versus the reference electrode at *T* = 20 °C.

### 4.3. SPR Sample Preparation

Self-assembled monolayers (SAMs) were formed from beta mercaptoethanol (βME) (Sigma-Aldrich, St. Louis, MO, USA) and WC14, which was synthesized, purified, and characterized in house, as reported previously in the Supplementary Material of Ref. [20]. SAMs were formed on thin Au layers (~460 Å) deposited by direct current magnetron sputtering (Auto A306; BOC Edwards, Crawley, West Sussex, UK) on glass slides coated with an ~5 Å thick Cr adhesion layer. The Au films typically had a uniformity of thickness across the surface of 3% or better, as determined by ellipsometry and transmission UV/VIS spectroscopy.

Mixed SAMs [20,28] were prepared by exposing magnetron-sputtered Au films to solutions of 20-tetradecyloxy-3,6,9,12,15,18,22- heptaoxahexatricontane-1-thiol (WC14):β-ME (3:7 mol/mol, 0.2 mM total concentration) in 99.5% ethanol for >12 h [20]. Because of the short hexa(ethylene oxide) tether, these SAMs and completed tBLMs incorporate hydrated sub-membrane layers that are only 15 Å [28]. The tBLMs were formed through a rapid solvent exchange procedure [20,28] where 20 μL of ~10 mM DPhyPC in ethanol is added to a 6 mm diameter cylindrical cell (total volume 1 mL) fixed to the SAM covered Au electrode surface with an o-ring and subsequently replaced with an aqueous buffer. This rapid solvent exchange procedure leads to the formation of complete and electrically insulating bilayers [20,28]. After tBLM formation, the surface was washed with pure water to remove excess phospholipids adsorbed on the tBLM layer. The tBLMs used here had residual specific conductance <3 mS·cm^2^. After tBLM formation, SPR, and EIS measurements were performed.
